# Sport-related concussion research agenda beyond medical science: culture, ethics, science, policy

**DOI:** 10.1136/jme-2022-108812

**Published:** 2023-03-03

**Authors:** Mike McNamee, Lynley C Anderson, Pascal Borry, Silvia Camporesi, Wayne Derman, Soren Holm, Taryn Rebecca Knox, Bert Leuridan, Sigmund Loland, Francisco Javier Lopez Frias, Ludovica Lorusso, Dominic Malcolm, David McArdle, Brad Partridge, Thomas Schramme, Mike Weed

**Affiliations:** 1 Department of Movement Sciences, KU Leuven, Leuven, Belgium; 2 School of Sport and Exercise Sciences, Swansea University, Swansea, UK; 3 Bioethics Centre, University of Otago, Dunedin, New Zealand; 4 Department of Public Health and Primary Care, Leuven, Leuven, Belgium; 5 Global Health & Social Medicine, King's College London, London, UK; 6 Department of Political Sciences, University of Vienna, Wien, Austria; 7 Institute of Sport & Exercise Medicine, Dept of Exercise, Sport & Lifestyle Medicine, Facuty of Medicine & Health Science, Stellenbosch University, Stellenbosch, South Africa; 8 IOC Research Center, Stellenbosch, South Africa; 9 Centre for Social Ethics and Policy, University of Manchester, Manchester, UK; 10 Centre for Medical Ethics, University of Oslo, Oslo, Norway; 11 Centre for Philosophical Psychology, University of Antwerp, Antwerpen, Belgium; 12 Department of Sport and Social Sciences, Norwegian School of Sports Sciences, Oslo, Norway; 13 Department of Kinesiology and Rock Ethics Institute, Penn State University, University Park, Pennsylvania, USA; 14 Departament de Psicologia Social, Universitat Autonoma de Barcelona, Barcelona, Spain; 15 School of Sport, Exercise and Health Sciences, Loughborough University, Loughborough, UK; 16 School of Law, University of Stirling, Stirling, UK; 17 Faculty of Medicine, The University of Queensland, Saint Lucia, Queensland, Australia; 18 Department of Philosophy, University of Liverpool Faculty of Humanities and Social Sciences, Liverpool, UK; 19 Centre for Sport, Physical Education & Activity Research (spear), Canterbury Christ Church University, Canterbury, UK

**Keywords:** ethics- medical, ethics, ethics- research, cultural diversity, decision making

## Abstract

The Concussion in Sport Group guidelines have successfully brought the attention of brain injuries to the global medical and sport research communities, and has significantly impacted brain injury-related practices and rules of international sport. Despite being the global repository of state-of-the-art science, diagnostic tools and guides to clinical practice, the ensuing consensus statements remain the object of ethical and sociocultural criticism. The purpose of this paper is to bring to bear a broad range of multidisciplinary challenges to the processes and products of sport-related concussion movement. We identify lacunae in scientific research and clinical guidance in relation to age, disability, gender and race. We also identify, through multidisciplinary and interdisciplinary analysis, a range of ethical problems resulting from conflicts of interest, processes of attributing expertise in sport-related concussion, unjustifiably narrow methodological control and insufficient athlete engagement in research and policy development. We argue that the sport and exercise medicine community need to augment the existing research and practice foci to understand these problems more holistically and, in turn, provide guidance and recommendations that help sport clinicians better care for brain-injured athletes.

## Introduction

 In 2017, the Berlin Concussion in Sport Group Consensus Statement (CiSG CS) was published alongside the Concussion Assessment Tool 5, the SCAT5 and Child SCAT5, ‘with the objective of offering a simple, clear message with tools that equip the practitioner to diagnose and manage concussion in various different sports’.^1^ Despite the plethora of professional and scientific guides, consensuses and reviews of concussion and other head injuries in sport, it is undoubtedly the leading professional locus of state-of-the-art knowledge for sport physicians (SPs), healthcare professionals (HCPs) and other stakeholders such as athletes or, more specifically, athletes when considered as patients (AP), event organisers and sport governing bodies. Despite the undeniable development in sophistication of the CSs, the CiSG, there has been a plenitude of criticisms that range from challenges concerning the narrowness in scope of the research, to ethical problems arising from various sources including conflicts of interest (CoIs), the reductionist or exclusive character of particular methodologies, and the problem of how to amalgamate highly diverse sources of evidence. The present article is the product of a symposium held at KU Leuven, 10 September 2022–21 September 2022 that sought—from multidisciplinary perspectives (medical ethics, genetic ethics, philosophy of science, policy studies, political sociology, professional ethics, sport ethics, sport law, sport sociology and sport medicine ethics) to broaden the scope of the CSs and make progress on conceptual, ethical and scientific challenges by establishing a multidisciplinary concussion research agenda beyond biomedical science and practice.

### Whose expertise: which research?

CiSG members have pointed to relevant expertise as important to the success of process that results in each iteration of the CS.^2^

Every 4 years researchers and clinicians with expertise in SRC (sports-related concussion)^i^ are brought together to summarise the published literature and provide updated, evidence informed recommendations regarding the evaluation and management of concussions… Authors have been selected based on their research and clinical expertise in the area of concussion.^2^

The coalescence of experts within this nascent medical field has happened relatively rapidly. The previous lead of the CiSG recalled that when they began their career as a team doctor even very experienced sports-medicine practitioners understood almost nothing about concussion, and in the years prior to the creation of the CiSG ‘any management recommendations were purely anecdotal.’^3^ Given how widely disseminated and influential the recommendations of the CiSG have become, it is pertinent to think more deeply about questions related to the nature of expertise and the power of experts. Exactly who is an expert, and why (when it comes to concussion management)?

Philosophical accounts of expertise reveal both epistemic conditions (ie, advanced knowledge or competency), but also social acknowledgement.^4^ Having ‘expertise’ is distinct from being credited as an ‘expert’, which is a quality ascribed to someone who fills a certain social role.^5^ The latter is not merely acknowledged but conferred.^6^ Expert status thus depends on recognition or legitimation, in socially, culturally and politically constructed ways. Recognition of expert status is therefore contingent on power dynamics. Being an expert is not simply a matter of what one knows, but also a matter of the processes that establish: who is ‘recognised’?; who is given a platform?; how they got there?; who influences/controls what is and is not researched?; who pays the bills?; which values and norms are evoked?; and of course, which voices are not included?. These questions are relevant to any critical examination of how expertise and expert status have informed all the iterations of the CSs, given that these documents provide an authoritative platform validating the expertise they claim.

How might recognition of these processes inform criticism of the CSs or challenge the claim that it represents the best synthesis of the current evidence? The composition of the group, the agenda, the scope, the implementation and the influence are contingent on structural and political factors. But what is considered to be expertise in this area can, in some ways, be constructed by entities with much to gain (or lose). It is, therefore, critical from an ethical, scientific and policy making perspective that the CiSG Scientific Committee and the pool of experts it draws on is selected from a broad pool of experts. Whether a group comprised of different concussion experts would reach the same ‘consensus’ is a moot point. This issue leads immediately to the concept of ‘consensus’ itself. How much agreement is required? Is this a mere quantitative question (eg, 80% of the conference audience) or a qualitative one (to what extent can experts still reasonably disagree about scientific and policy matters that fall within the purview of the CS while remaining a Consensus)? And finally, what is critical here for confidence in the CS is the matter of public awareness: how are the groups, selection processes and their workings made transparent?

Having drawn attention to the social and political dimensions of expertise it is necessary also to draw out the ethical corollaries. The motives of powerful groups directly or indirectly facilitate expert status subjecting CSs to many interest groups, including large professional sporting leagues, private companies and funding bodies.^7^ Declarations of competing interests in previous CSs have been varied in terms of their comprehensiveness, and this aspect of the CiSG’s activities is very much a work in progress.^ii^ How is this to be reconciled given that their recommendations are intended to be practical and independent? A declaration of interest is not necessarily a sufficient response to potential or perceived CoIs. The challenge is that leaders of the concussion movement may have legitimate claims to expert status and CoIs. It may ultimately be that contributing to the powerful consensus positions is incompatible with holding leading positions with other stakeholders whose influence could—or could be seen to—motivate bias or partiality. Others may argue that this is a case of throwing the baby out with the bathwater. Further discussion of professional ethics and academic integrity is required here.

### Whose research, with and for whom?

Many sports generate a substantial proportion of their revenue from the elite level, yet the long-term maintenance of interest in the sport requires participation at all levels from the recreational to the elite. Sports federations also have a prudential interest in mitigating known or perceived health risks that can potentially reduce present or future participation by children or adults. Moreover, they have an ethical interest in maintaining a relation of reciprocity with the grassroots participants.^8^ Sports federations are the ‘dominant self-regulators’ in sport,^9^ and this status generates a duty of care or diligence.^10^ This extends directly to an obligation to initiate, facilitate and fund independent knowledge generation, that is, research about serious sports-related injuries. These prudential (ie, self-interested) and reciprocity-based reasons also support obligations to initiate, facilitate and fund research. The ability of a particular sports federation to discharge these research obligations depends on the resources available, but those who can fund should, and of course, their decisions should be guided by impartial state-of-the-art reviews and CSs.

Moreover, it is increasingly recognised in biomedical research that research projects improve if patients are involved in choosing the research questions and study design. This is usually termed patient and public involvement and engagement and is now a requirement imposed by many funders. Similarly, concussion research in sport is likely to be better and more relevant if, for instance, active athletes, coaches and officials are involved in the form of APIE (athlete participation, involvement and engagement). The reasons for making APIE an integral part of research are fourfold. First, it is likely to promote buy-in from athletes, coaches and officials to the research itself, but also to the implementation of any interventions flowing from the research. Second, implementation science shows convincingly, that unless the end-users of knowledge are involved in the knowledge production at all stages, obstacles to implementation are likely to be overlooked.^11 12^ Third, there are aspects of being an active athlete that enables more immediate access to knowledge and experience than most researchers have. This is not to say that other people cannot get access to this knowledge, but recognising that it would require work to get it, and that it can only be obtained by asking the right questions. Philosophically, this can be explicated as a moderate form of standpoint epistemology, that is, the idea that those who are engaged in a practice or are in a particular social position have privileged access to knowledge about that practice.^13^ In this sense, HCPs cannot claim convincingly to speak for their APs. Fourth, the consequences of concussion that are of primary interest to athletes may be slightly different from those of primary interest to researchers and HCPs, just as authors of the CS cannot necessarily claim to speak for HCPs practising across all levels of sport. This may especially be true in relation to the more acute neurological symptoms following a concussion, or in relation to the implications of concussion incidents for amateur athletes in relation to work or education.

### Which evidence counts?

Talk of consensus in concussion research leads inexorably to scientific questions around methodology, but also crucially, to normative discussions around which methodologies are privileged and why. Research into concussion, concussion prevention and concussion management has hitherto aimed at finding causal relations (how can they be accurately diagnosed; what are the long-term effects; how can the effects be mitigated, etc). Establishing the quality of evidence that contributes to a consensus has been open to considerable dispute across previous CSs (and their underlying processes).

First, the various SCs have used the PROSPERO protocol. In line with evidence-based medicine recommendations,^14^ systematic reviews would usually focus on a meta-analysis of randomised controlled trials (RCTs) are focused on, meaning that research arising from case studies or case series, such as those for chronic traumatic encephalopathy (CTE), which have been evaluated using the Bradford Hill criteria,^15^ have hitherto not been incorporated into the CSs. RCTs are widely regarded as the gold standard for causal research in evidence-based medicine. In addition, systematic reviews involving meta-analyses of RCTs are sometimes called the platinum standard; an honorific title not without its critics.^16 17^ Yet RCTs are not always feasible due to a variety of reasons: ethical (eg, deliberately inflicting concussions in humans even with their consent is unlikely to pass any kind of ethical review board) and practical (eg, how to keep participants blinded with respect to their assigned group?). Many alternative methodologies exist, on humans (case–control, cohort, etc), on animals (laboratory RCTs), in vitro (mechanistic studies on, eg, brain tissue), and in silico (computer simulations). Within evidence-based medicine, these are typically considered of lower quality (see, eg, the Oxford Centre for Evidence Based Medicine (OCEBM) levels of evidence).^14^ But when faced with the dilemma to either (1) ignore scientific evidence beneath RCTS (cf. the purely anecdotal management recommendations mentioned above—even where promoted by experts) or (2) settle for a combination of alternative methodologies, the second option definitely should be preferred. Naturally, an appropriate justification for the use of any methodology in a particular context should be provided. Second, where one’s primary sources are methodologically homogeneous (eg, where all are studies based on RCTs using similar protocols), systematic reviewing and meta-analysis are relatively straightforward. Yet this is not the case in concussion science. Unsurprisingly, amalgamating strongly diverging/heterogeneous primary studies is much more difficult.

The integration of heterogeneous evidentiary bases does not represent an insuperable problem. A similar problem plaguing cancer epidemiology has been addressed by the International Agency for Research on Cancer (IARC) during the past six decades.^18^ Due to ethical considerations, few if any human-based RCTs identifying carcinogenic hazards exist. For most purported hazards, however, there do exist both observational (non-experimental) epidemiological studies in humans, and laboratory RCTs on animals as well as mechanistic studies. IARC combines these diverse streams of evidence in workgroup meetings aimed at peer review and consensus. This procedure could, with appropriate revisions, serve as a source of inspiration for the CiSG, though it could be argued that other medical conditions might serve as better analogues, if more pluralistic approaches have been found to be applicable and efficacious.

### Genetic-ethical considerations

How might genetic science contribute to concussion science and care? Various studies have investigated the role of genetic factors in (A) predicting the risk of concussion and traumatic brain injury^19^ and (B) predicting recovery and management from concussion.^20^ Nevertheless, promises of identification of those athletes/APs at greater risks of poor outcomes or those of improved diagnosis, therapy and prevention by such genomic susceptibility testing should be analysed critically. For many associations, the predictive value is low and more studies are necessary to validate findings.^21^ The vast majority of tests for common disorders are still purely research-based and have not received any formal evaluation in terms of clinical validity and utility.^22^

Various ethical concerns follow the introduction of such tests. Although genetic susceptibility information has a probabilistic nature, this type of information is often interpreted with a sense of determinism and by reduction of an individual to its genetic characteristic^23^ A risk stratification that puts someone at low risk of concussion (because of behavioural traits) is based on a simplistic causal relationship between genetic variants associated with cautious behaviour and avoidance of concussion. This might also create stigmatisation and labelling, privacy concerns, and inappropriate stratification. One might also be concerned with inappropriate use by insurers, medical professionals, or coaches when making decisions regarding return to play (RTP).^24^Moreover, as testing for concussion might also include variants in the APOE gene,^25^ it might, as a byproduct, disclose an ‘incidental’ increased risk for Alzheimer disease.^26^ This opens various other problems regarding counselling, informed consent, family risk, right not to know, insurance, etc. The prospects of genetic medicine in the area of concussion look, at this juncture, limited. Therefore, the CiSG Scientific Committee ought seriously to consider a public statement as to the utility of direct-to-consumer genetics tests for concussion.^27^

### Ethical framework for management of concussion: beyond respect for autonomy

The 2017 CS frames postbrain trauma situations as involving two independent agents, an AP and an SP/HCP, whose primary interest is the protection and promotion of health. Brain trauma management protocols based on dyadic clinical frameworks have successfully provided APs with greater protection. Remove-from-play protocols insulate APs from some of the pressures to continue playing after suffering trauma, and information and awareness programmes help APs and sport medicine professionals better understand the severity of head injuries. Arriving at more robust and ethically defensible RTP protocols has clearly been one of the successes of the 2017 CS process. Nevertheless, all such frameworks face limitations. Health-related decisions can hardly be understood as simply involving two agents who agree on the primacy of clinical criteria and prioritise health-related interests. This framing obscures two critical elements: (A) APs make (more or less informed) choices in complex contexts and (B) APs, often as a result of contextual elements, deprioritise health-related concerns.

SRCs incorporate complex processes originating from myriad agents and factors. APs’ decision-making contexts comprise specific individuals (eg, relatives, teammates, coaches), institutions (eg, team, family) and policies (eg, contract, legal frameworks), all of which have rich histories and traditions (consider the self-sacrifice culture in high-contact sports like Rugby Union and American Football).^28^,^31^ All these elements can affect APs’ decision making. Moreover, all AP-clinician encounters arise in specific contexts. Compare an adult AP playing football in a prestigious US university and a female amateur rugby player competing in a community-based tournament. Failing to account for contextual elements is likely to undermine the effectiveness of concussion management protocols for at least two reasons. First, the protocols misalign with APs’ experiences, placing unworkable expectations on APs and issuing unfeasible recommendations; second, management protocols can only be effective if they follow clinicians’ recommendations.

The CS recognises that ‘treatment should be individualised and target specific medical, physical and psychosocial factors’^32^ and recommends approaching brain trauma incidents through multidisciplinary lenses. This typically means different disciplines within healthcare and medicine, not beyond it and in doing so, prioritises biologically based factors at the expense of others. For example, sociocultural elements are mentioned once, at the end of the ‘Prevention’ section, but the document leaves unexplained the relevance of these factors—other than noting that they ‘play a significant role in the uptake of any injury-prevention strategy.’^32^

Within the medical or healthcare domain, all SPs and HCPs are aware that informed consent requires mental capacity (especially reasonable comprehension of sufficient information) as well as voluntariness and the absence of coercive forces. Concussion management should always follow these norms and rules, adapting them specifically to their AP. The use of the SCAT test can at best serve as a proxy for mental capacity. Some cases (eg, children/adolescents/athletes with intellectual impairments) will clearly require special measures—even for the implementation of SCAT5, let alone other significant decisions. Also, relevant non-paternalistic reasons, such as the protection of other players, may require removing a player. In general, however, efforts must be made to involve players in decision making. This might involve advance directives for situations of unestablished (or unknown) mental capacity. Justified policies must go beyond medical facts. Individuals’ preferences and especially assessment of medical considerations differ widely. In contradistinction to the perspectives of many SP, the promotion and protection of health cannot generally be presumed to be athletes’ main, or even only, concern.^33^

Sufficiently informing players requires openness about possible postconcussion long-term effects. Especially important is transparency about the difference between risks and uncertainties, as these must be assessed differently.^34^ Risks resulting from playing sports can be individually weighed because the relevant facts, including probabilities of brain injury, are sufficiently established. In contrast, evidence concerning the nature of pertinent chronic health conditions and the role brain injuries may have in their progression is weaker.^35^,^38^ This uncertainty, and accordingly, the problem for individuals to weigh their options, is not merely due to lack of knowledge about causal connections between repeated concussions and chronic conditions. Rather, the problem stems from the population-specific character of extant evidence. Relevant studies examine particular groups of people, such as elite athletes, and perhaps compare them with other groups in specific respects. Consequently, potential health impacts also apply to these abstract group categories. Epidemiological studies result in statistical generalisations.^39^ Individual players ignore the specific probability of long-term harm to weigh against the benefits of play. Thus, they lack adequate information. In such a situation, players need support to make autonomous choices. For instance, they can be provided with information about natural frequencies of chronic brain diseases in specific populations. Making people health literate is an established goal of public health measures to broaden their medical perspective and help them recognise ethical considerations.^40^ Reflecting on autonomy, philosophers have attempted to devise formulae to safeguard individual decision making without leaving people alone in the formidable task of making autonomous decisions. For instance, the idea of scaffolding autonomy has been discussed and would be one idea to build on when considering the autonomy of APs in situations of relative uncertainty.^41 42^

### Considerations for children and adolescents

A paucity of research exists into concussion in children, especially younger children. Children are vulnerable in virtue of their emerging capacity to make complex decisions or take actions that influence their lives, meaning that parents or other proxy decision-makers are required. Furthermore, children are potentially physiologically vulnerability due to growth and development of different systems at different ages.^43^ These considerations must be factored into welfare discussions, specifically in relation to brain injuries. Children may also not be able to identify SRC for themselves, and there may not be trained medical staff at their sporting events able to provide care.

The presence of trained staff and infrastructure for dealing with concussion is more likely at the elite level. This point applies to many issues concerning SRC, and so the development of the Concussion Recognition Tool (CRT5), designed for use by all stakeholders, is most welcome.^44^ Nevertheless, at the recreational level, parents and coaches may not be familiar with CRT5 use. Even if used correctly, a range of ethical considerations arise. Children are dependent on their parents to access medical care, which may not be available or affordable. Where medical services are available, questions remain about proxy decision-making. Parents at all sporting levels may not always act in the best interests of their child. Parents may not believe the child regarding their symptoms, wish for a faster RTP (especially in higher levels of sport competition), or not acknowledge or even be aware of the process of recovery or the risks posed by early RTP. Equally, they may simply be overprotective based on a failure to understand these protocols.

Equally, coaches may also not recognise the significance of a (sub)concussive episode, insisting on a timeline for RTP that is unrealistic, especially in the absence of reliable biomarkers. Some will recover in days to weeks, while up to 30% of children and youth may have on-going symptoms 30 days following concussion.^43 44^ The literature in this area continues to evolve. The SP or HCP may struggle to balance the needs of the AP with the expectations of the coach and the child’s parents, especially when seeking parental consent.

Given that concussions are more likely or severe in contact/collision sport, the judgement around a child’s participation must entail an estimation of likelihood and severity of the potential harm.^45^ Two possible frameworks for such judgements should be considered. The best interests (BI) framework (which clinicians might typically use) aims to maximise a child’s interests, based on a trade-off among their various interests. BI judgements are unlikely to support participation where concussion is both likely and severe. It is, however, notoriously difficult to determine what is in a child’s BI especially when uncertainty exists. Moreover, and more generally, no society requires all decisions to be in the child’s BI,^1^ instead, parents and the State are only required to ensure that a child receives a certain level of care.^46^

An oft-cited alternative to BI is the ‘right to an open future’ (ROF)^47^—any decision made for a child must not limit the child’s future options. ‘The principle holds that children possess a unique class of rights called rights in trust—rights that they cannot yet exercise, but which they will be able to exercise when they reach maturity’.^48^ A concussion with persisting symptoms may limit the child’s future decision-making capacity. ROF is especially applicable to concussion in children as they may experience long-term sequelae from significant or repeated incidents. Although less restrictive than BI, ROF judgements struggle to balance the opportunity costs involved in conflicting potential future lives.^49^ When making decisions on behalf of a child all emerging evidence must be considered in line with the precautionary principle and in association with BI and/or ROF.

### Race and risk for adverse outcomes following (repetitive) head injuries

Recent data on active and former American football players who self-identify as black point to increased risk factors for long-term adverse neurological outcomes following repetitive head injuries.^50^,^52^ Several aspects of this standpoint warrant critical scrutiny. First, self-identified racial identity depends on several kinds of psychological, cultural and social factors not always explicitly recognised in the methodology.^53^,^55^ The observed associations between self-identified racial identities and adverse outcomes following TBI in the recent data on former football players who self-identify as black could be explained only with the psychosocial, economic and cultural factors for which self-identified race is used as a proxy.

Many studies^56 57^ demonstrate that perceived racial discrimination through a phenomenon known as ‘allostatic effect’ have an adverse effect on health in general through epigenetic stress mechanisms and leads to predispositions to a variety of multifactorial diseases.^58^ Negative feedback loops triggered by using concepts such as self-identified race as a proxy for increased risks of adverse outcomes reinforce stereotype threats, leading to athletes ruling themselves out from sports for which they think have no predisposition, or perform at a diminished level.^59 60^ Therefore, using self-identified race as a proxy for the multitude of factors associated with adverse health outcomes is neither epistemologically, nor ethically justified.^53^ Notwithstanding repeated calls by to the contrary,^61 62^ we argue that it is problematic that NIH requires the use of self-identified race in research, as it leads to the biological reification/reinscription of a social concept.

More research is needed to investigate the psychosocial, economic and cultural factors which can lead to adverse neurological outcomes following concussion. Robust social epidemiological studies of health disparities exist in other contexts, which should be taken as trustworthy exemplars and applied to this context.^57 58 63^

### Gender and SRC

The growing body of research on SRC and gender suggests that females experience concussion more frequently, experience more severe symptoms and take longer than males to recover.^64^,^67^ These studies correct the historic male bias in SRC research, which led previous CSs to rely on evidence drawn from study samples that were overall 80.1% male.^68^ Also concerning is the dominance in this literature that attributes patterns of incidence and impact to physical (eg, visual awareness, neck strength) and physiological (eg, hormonal, brain structure) rather than social factors (eg, reporting behaviours). The prioritisation of physiological and physical factors is in contrast to the dominant sport injury models, which either propose a multifactorial aetiology^69^ or target individual behavioural prevention initiatives.^70^ The promotion of different explanatory paradigms to explain male and female SRCs replicates the historical gender bias in sports science medical research^71 72^ that has recently come under increasing scrutiny.^73^ The policy recommendations that currently have been proposed on the basis of this research call for sex-differentiated risk mitigation, treatment and athlete surveillance; yet, these should all be rejected on ethical and social justice principles. A preferable approach is to ensure that activities are revised to be safe for all participants rather than risk the exclusion of more vulnerable populations. Such an approach would bring the response to SRC into alignment with the principles of occupational health,^74^ and thus is likely to be more broadly socially acceptable.

### Accommodating difference and disability in SRC science and care

Para athletes, a smaller class than athletes with disabilities who compete/participate in activities not regulated by the International Paralympic Committee, can be broadly considered to fit into one of three major diagnostic categories: visual impairment, physical impairment and intellectual impairment.^75^ Of and within these three categories, athletes who are visually impaired, athletes with limb deficiency, athletes with cerebral palsy and athletes with spinal cord injury constitute the largest Para athlete groups.

Concussion in Para sport (CIPS) has largely been unrecognised and under-reported.^76^ Nevertheless, head, neck and facial injuries (often observed as loss of consciousness or subsequent ataxia) have been documented in both summer and winter Games settings.^77 78^ From these data, several sports have been identified as of potential higher risk of SRC, particularly those with speed, collision and contact as inherent risk factors. These sports include blind football and alpine skiing, as well as certain cycling, equestrian, and track and field events.

Aspects related to these inherent sport risks in the setting of impairment(s) of APs increases the complexity and uncertainty regarding aspects of SRC recognition, diagnosis, management and RTP decisions, all of which make this population further vulnerable. Specific examples of these vulnerabilities include the fact that Para APs have already adapted to congenital or acquired impairments, and a recognition of this is challenging to accommodate in clinical and team management discussions. AP disclosure to the SP or HCP may vary significantly because of this. Second, the fitness-for-purpose of the SCAT5 has not been significantly evaluated by researchers across a range of Para-sport categories. It is also known that baseline testing scores are sometimes different in the Para AP population.^56^ Third, it is not fully clear how adaptations related to SRC management should be implemented, for example, the concept of rest is different to a wheelchair-using AP who is expending significant energy as part of daily living.

A greater understanding of existing knowledge gaps and attitudes towards SRC among para APs, coaches and medical staff, are topics only recently receiving sustained attention and research focus in the para sports medicine arena, as efforts to work together with all stakeholders to reduce SRC gather momentum.^79^ The CiSG group should consider, along with the newly formed CIPS, how the resulting data can be effectively integrated.

### Intervening in sport regulation change

Rule adaptation and practice-related changes within sports competition and training are almost inevitable. Within sports, rules can be categorised as either constitutive or regulative.^80^ The distinction has proved fertile in analyses of the rule structures in sport.^81^ Constitutive rules create an activity the existence of which is logically dependent on the rules. The rules make sense only in the practice they define. Regulative rules regulate pre-existing activities whose existence is independent of the rules. What are the implications of this distinction in the context of SRC?

As indicated in previous CSs, most preventive rule adaptations focus on concussion management itself.^82 83^ Perhaps, however, the main cause of concerns about SRC lie in the nature of sport itself, which expose APs to SRC risks. One radical solution is to reject the constitutive rules in sports with concussion risk assessments above a set level. For example, the World Medical Association previously recommended a ban on boxing.^84^ Less radical measures focus on the modification of constitutive rules (eg, body checking in Canadian youth ice hockey that led to reduced injury and SRC rates).^85^

For a holistic understanding, biomedical approaches to SRC should be augmented by, or framed within, ‘thicker’ sociocultural understandings. This requires nuanced insight and understanding of the standards of excellence in particular sports, including how safety-based concerns can be evaluated against sports with foreseeable head injuries without eradicating, where at all possible, their defining internal goods, values or excellences.^80^ This contextualissation of SRC risks enables a distinction between relevant and non-relevant risks. Some SRC risks (eg, in boxing and downhill skiing) are intimately linked to the standards of excellence of these sports. In this sense, they are ‘relevant’ though not necessarily ethically acceptable. The constitutive rules of boxing encourage the exposure of opponents to increased SRC risks and could be considered unacceptable,^84^ whereas SRC risk in downhill skiing is considered an unavoidable element of skiing skills and may be considered acceptable (up to a certain level). Other forms of risk (eg, failure to use protective headgear in boxing and downhill skiing) are not connected to sporting standards of excellence and can be deemed both non-relevant and non-acceptable. What is required is the appraisal of both risks and the defining excellences and goods of the activity to locate pressure points and to debate them with appropriate medical, philosophical, regulatory and sociocultural expertise.

### A pragmatic legal response to SRC

Legal structures have little to offer as a global one-size-fits-all solution to SRC concerns. Statutory responses unfairly shift responsibility onto coaches and match officials; personal injury law demands the near-impossibility of showing both a breach of the duty of care and establishing causation in respect of conditions that can be incredibly difficult to detect in sports settings; and neither approach facilitates recognition and response to a condition which might not present itself until several days later.^86^ Further, if one accepts that SRC is a global phenomenon that, by definition, demands global responses, then whether at the elite or grass roots level, and whether the concern is with adults or children, it is not ‘law’s territory’.

There has been much discussion of athletes’ workplace status as a key issue in determining the applicability of SRC-related rights and obligations arising under (eg) national health and safety laws, disability and other forms of discrimination, whistleblowing and vicarious (employer) liability. But in some individual sports especially, athletes are often regarded as self-employed in some countries, while, under the same conditions, they would have worker or employee status elsewhere. In the English case of Varnish v British Cycling UKEAT/0022/20/LA, for example, an Olympic Cyclist was held to be self-employed and was thus unable to bring a sex discrimination claim, while Northwestern University and College Athletes Players Association Case 13-RC-121359 explains why US college football players cannot unionise under national law. By contrast, many jurisdictions have legislation in the form of Sports Acts,^87^ which confirm the employment status of elite athletes and that workplace status can facilitate union membership or other forms of collective representation as well as provide access to the remedies noted above. No conversations about SRC at the elite level should take place without high-level engagement with athlete unions or other player representatives, but if SRC-related rights and obligations are rooted in domestic law, they will inevitably vary across jurisdictions as much as they vary across disciplines. That cannot be reconciled with the evident need for a global approach to the problem.

Protocols, which either apply globally to a particular sport or to every sport within a particular country, have far more to offer than reverting to legal remedy. Much can be learnt from the Scottish experience with If in Doubt, Sit Them Out, a national SRC strategy launched in 2015 with the support of all sports and the Scottish government, employing a precautionary approach, mandating a minimum level of education, awareness and accountability regardless of the level of participation, supporting removal from play where decision makers are in any doubt.^88^ The strategy is not without its weaknesses, notably in broader applicability. While reliance on volunteers is a key feature of the European Model of Sports,^89^ so much so that grass roots participation could not function without them,^90^ a key difficulty with If in Doubt is that volunteers, employees and workers are expected to understand and implement advice on detection and response which sports organisations, the government and medical experts had taken several years to develop. The usefulness of if in doubt is currently limited by the unrealistic expectations it places on stakeholders and, crucially, the lack of awareness among those groups. In some ways, it is a failed performative,^91^ but it provides a starting point that individual countries and global sports can build on; it has far more to offer than recourse to law, helps overcome some inevitable jurisdictional differences and gives agency to participants and other sporting stakeholders that legal strategies would deny.

### From SRC research to policy development

The role of science is to set out what the evidence says, whereas the role of policy is to consider whether the evidence merits a policy response, given the policy purpose. Effective policy for SRC must have a clear and credible policy purpose. The CiSG consensus statements^92^ aim ‘to provide recommendations for the improvement of safety and health of athletes who suffer concussive injuries…for use by…people involved in the care of injured athletes, whether at the recreational, elite or professional level’. This sets a clear purpose: to mitigate the acute health impact of SRC episodes. Nevertheless, it is not credible for policy for SRC to be limited to mitigating acute health impacts of SRC episodes once they occur. Thus, a complementary policy purpose to reduce SRC incidence could be added to the purpose of the CiSG statements.

Policy arising from the CS must be informed by efficacy (what works in ideal controlled conditions) and effectiveness (what works in the real world) evidence that policy recommendations can achieve their policy purpose by the means proposed.^93^ Despite being informed by evidence from 12 systematic reviews,^2094^,^104^ and thus having assumptive ‘theoretical efficacy’, there is currently very limited postimplementation evidence for the effectiveness of the CS recommendations in relation to relevant policy purposes.

The CS includes discussions on prevention, risk reduction and ‘sequelae’ (long-term effects)^9295 105^,^107^ but the latter refer only to whether and what long-term effects exist. In the latest CS^94^ specific recommendations for the former, informed by a commissioned systematic review,^108^ are included but extend only to snow-sport helmets and rule enforcement for high elbows in soccer.

A more comprehensive policy purpose would be to improve long-term brain health outcomes, to which mitigating the acute health impact of SRC may or may not contribute, and towards which there is a clear role for a more extensive focus on incidence reduction. Debates about a demonstrable cause-and-effect relationship rest on discussions, highlighted earlier, concerning what evidence counts in relation to SRC, including long-term effects and conditions such as CTE. Nevertheless, the mere existence of the debate, and its prominence within concussion science, demands that a policy response be considered. In turn, greater cooperation between the various stakeholders could facilitate more credible SRC policy.^109^

## Conclusions: joined up research to accommodate ethical, cultural and governance reform

While the 2017 CS identifies education as ‘a mainstay of progress in this field’, progress does not match the expectations of campaigners.^110^ This has led to calls for a ‘cultural change’ regarding SRC policy relating to reporting behaviours, treatment compliance, attitudes, behaviours and social norms.^111 112^ As increasing multidisciplinary knowledge and more precautionary attitudes towards concussion have arisen, progress must be evaluated against greater attention to context: on the cultural and structural aspects of sport’s organisation of which sport medicine will play an important role.

At an organisational level, explicit identification of responsibility for the management of concussion should be encouraged at recreational levels. Responsibility should be accompanied by regulatory empowerment. For non-elite athletes, consideration should be given to the responsibility for decisions to remove participants from play, the ongoing monitoring of symptoms, and supervision of RTP. The lower the skill levels/qualifications available, the wider that responsibility should be shared, following broad principles of precaution. Care should be taken to enable concussed athletes to remain both integrated and integral to sports teams/organisations in the longer term, as this will encourage precautionary behaviours.

In medical terms, the introduction of independent medical assessments and diagnoses for concussion is to be welcomed. Further clinical autonomy, appropriately coordinated, across sports medicine should facilitate positive cultural change. Relevant mechanisms would consider recruitment and appointment procedures, ethical and legally sound protocols for managing information exchange between coaching and medical staff, and improved understanding of medical ethics within sports organisations.

We have attempted to demonstrate by example and argument how a broader multidisciplinary approach can augment and better situate biomedical research and clinical practice in SRC more holistically. With the SRC widely acknowledged as a form of global problem, now is not the time for HPCs, SPs and biomedical researchers to turn inwards. Mutually respectful and inclusive research-based dialogue is essential for the development of the SRC agenda.

## Data Availability

No data are available.

## Footnotes

Although there is no definitive label for the phenomenon of concussion in sport, Ssport R-related Cconcussion will be used throughout. This is in acknowledgement of the fact that the CiSG have adopted this label and because biomedical researchers and clinicians have typically tended to distinguish brain trauma into military blast, road trauma, and sport categories. Thanks to Gavin Davies for this clarification.

We acknowledge that measures in place for the 2022 Amsterdam conference and the resultant Systematic Reviews and CS itself have improved the state of affairs.
